# Processed pseudogenes acquired somatically during cancer development

**DOI:** 10.1038/ncomms4644

**Published:** 2014-04-09

**Authors:** Susanna L. Cooke, Adam Shlien, John Marshall, Christodoulos P. Pipinikas, Inigo Martincorena, Jose M.C. Tubio, Yilong Li, Andrew Menzies, Laura Mudie, Manasa Ramakrishna, Lucy Yates, Helen Davies, Niccolo Bolli, Graham R. Bignell, Patrick S. Tarpey, Sam Behjati, Serena Nik-Zainal, Elli Papaemmanuil, Vitor H. Teixeira, Keiran Raine, Sarah O’Meara, Maryam S. Dodoran, Jon W. Teague, Adam P. Butler, Christine Iacobuzio-Donahue, Thomas Santarius, Richard G. Grundy, David Malkin, Mel Greaves, Nikhil Munshi, Adrienne M. Flanagan, David Bowtell, Sancha Martin, Denis Larsimont, Jorge S. Reis-Filho, Alex Boussioutas, Jack A. Taylor, Neil D. Hayes, Sam M. Janes, P. Andrew Futreal, Michael R. Stratton, Ultan McDermott, Peter J. Campbell, ICGC Breast Cancer Group, Elena Provenzano, Marc van de Vijver, Andrea L. Richardson, Colin Purdie, Sarah Pinder, Gaetan Mac Grogan, Anne Vincent-Salomon, Denis Larsimont, Dorthe Grabau, Torill Sauer, Øystein Garred, Anna Ehinger, Gert G. Van den Eynden, C.H.M van Deurzen, Roberto Salgado, Jane E. Brock, Sunil R. Lakhani, Dilip D. Giri, Laurent Arnould, Jocelyne Jacquemier, Isabelle Treilleux, Carlos Caldas, Suet-Feung Chin, Aquila Fatima, Alastair M. Thompson, Alasdair Stenhouse, John Foekens, John Martens, Anieta Sieuwerts, Arjen Brinkman, Henk Stunnenberg, Paul N. Span, Fred Sweep, Christine Desmedt, Christos Sotiriou, Gilles Thomas, Annegein Broeks, Anita Langerod, Samuel Aparicio, Peter T. Simpson, Laura van ’t Veer, Jórunn Erla Eyfjörd, Holmfridur Hilmarsdottir, Jon G. Jonasson, Anne-Lise Børresen-Dale, Ming Ta Michael Lee, Bernice Huimin Wong, Benita Kiat Tee Tan, Gerrit K.J. Hooijer

**Affiliations:** 1 Cancer Genome Project, Wellcome Trust Sanger Institute, Genome Campus, Hinxton, Cambridgeshire CB10 1SA, UK; 2 Lungs for Living Research Centre, Rayne Institute, University College London, London WC1E 6JF, UK; 3 University of Cambridge, Cambridge CB2 0XY, UK; 4 Departments of Pathology and Oncology, Johns Hopkins Medical Institutions, Baltimore, Maryland 21205, USA; 5 Addenbrooke’s NHS Foundation Trust, Cambridge CB2 0QQ, UK; 6 Children’s Brain Tumour Research Centre, University of Nottingham, Nottingham NG7 2UH, UK; 7 Department of Pediatrics, Hospital for Sick Children, University of Toronto, Toronto, Ontario, Canada M5G 1X8; 8 Institute for Cancer Research, Sutton, London SM2 5NG, UK; 9 Dana-Farber Cancer Institute, Boston, Massachusetts 02215, USA; 10 Royal National Orthopaedic Hospital, Middlesex HA7 4LP, UK; 11 Peter MacCallum Cancer Centre, Melbourne, Victoria 3002, Australia; 12 Department of Pathology, Jules Bordet Institute, 1000 Brussels, Belgium; 13 Department of Pathology, Memorial-Sloan-Kettering Cancer Center, New York, New York 10065, USA; 14 Department of Gastroenterology, Royal Melbourne Hospital, University of Melbourne, Parkville, Victoria 3050, Australia; 15 National Institute of Environmental Health Sciences, Research Triangle Park, North Carolina 27713, USA; 16 UNC Lineberger Comprehensive Cancer Center, University of North Carolina, Chapel Hill, North Carolina 27599, USA; 17 Cambridge Breast Unit, Addenbrooke’s Hospital, Cambridge University Hospital NHS Foundation Trust and NIHR Cambridge Biomedical Research Centre, Cambridge CB2 0QQ, UK; 18 Department of Pathology, Academic Medical Center, Meibergdreef 9, 1105 Amsterdam, AZ, The Netherlands; 19 Department of Cancer Biology, Dana-Farber Cancer Institute, 450 Brookline Avenue, Boston, Massachusetts 02215, USA; 20 Department of Pathology, Brigham and Women’s Hospital, Harvard Medical School, 75 Francis Street, Boston, Massachusetts 02115, USA; 21 East of Scotland Breast Service, Ninewells Hospital, Dundee DD1 9SY, UK; 22 Department of Research Oncology, Guy’s Hospital, King’s Health Partners AHSC, King’s College London School of Medicine, London SE1 9RT, UK; 23 Institut Bergonié, 229 cours de l’Argone, 33076 Bordeaux, France; 24 Institut Curie, Department of Tumor Biology, 26 rue d’Ulm, 75248 Paris cédex 05, France; 25 Institut Curie, INSERM Unit 830, 26 rue d’Ulm, 75248 Paris cédex 05, France; 26 Department of Pathology, Jules Bordet Institute, 1000 Brussels, Belgium; 27 Department of Pathology, Skåne University Hospital, Lund University, SE-221 85 Lund, Sweden; 28 Department of Pathology, Oslo University Hospital Ulleval and University of Oslo, Faculty of Medicine and Institute of Clinical Medicine, 0450 Oslo, Norway; 29 Lund University, Division of Oncology, Department of Clinical Sciences, Lund University Cancer Center at Medicon Village, Lund SE-223 81, Sweden; 30 Blekinge Hospital, Department of Pathology and Cytology, SE-371 85 Karlskrona, Sweden; 31 Translational Cancer Research Unit, GZA Hospitals St Augustinus, 2610 Antwerp, Belgium; 32 Department of Pathology, Erasmus Medical Center, 3015 Rotterdam, The Netherlands; 33 The University of Queensland, School of Medicine, Herston, Brisbane, Queensland 4006, Australia; 34 Pathology Queensland: The Royal Brisbane & Women’s Hospital, Brisbane, Queensland 4029, Australia; 35 The University of Queensland, UQ Centre for Clinical Research, Herston, Brisbane, Queensland 4029, Australia; 36 Department of Pathology, Memorial Sloan-Kettering Cancer Center, New York, New York 10065, USA; 37 Centre Georges-François Leclerc, 1 rue du Professeur Marion, 21079 Dijon, France; 38 0nstitut Paoli Calmettes, Department of Biopathology, 232 Boulevard Sainte Marguerite, 13009 Marseille, France; 39 Centre Léon Bérard, Lyon, France; Université Claude Bernard Lyon1—Université de Lyon, 69008 Lyon, France; 40 Department of Oncology, University of Cambridge and Cancer Research UK Cambridge Research Institute, Li Ka Shin Centre, Cambridge CB2 0RE, UK; 41 Dundee Cancer Centre, Ninewells Hospital, Dundee DD1 9SY, UK; 42 Erasmus MC Cancer Institute, Erasmus University Medical Center, 3015 Rotterdam, The Netherlands; 43 Radboud University, Department of Molecular Biology, Faculty of Science, Nijmegen Centre for Molecular Life Sciences, 6500 HB Nijmegen, The Netherlands; 44 Department of Radiation Oncology, Radboud University Medical Centre, 6500 Nijmegen, The Netherlands; 45 Department of Laboratory Medicine, Radboud University Medical Centre, 6500 Nijmegen, The Netherlands; 46 Breast Cancer Translational Research Laboratory, Institut Jules Bordet, Université Libre de Bruxelles, 1000 Brussels, Belgium; 47 Universite Lyon1, INCa-Synergie, Centre Leon Berard, 28 rue Laennec, 69008 Lyon, France; 48 Department Experimental Therapy, The Netherlands Cancer Institute, Plesmanlaan 121, 1066 CX Amsterdam, The Netherlands; 49 Department of Genetics, Institute for Cancer Research, The Norwegian Radium Hospital, Oslo University Hospital, O310 Oslo, Norway; 50 Department of Molecular Oncology, BC Cancer Agency, 675 W10th Avenue, Vancouver, British Columbia, Canada V5Z 1L3; 51 The Netherlands Cancer Institute, Division of Molecular Carcinogenesis, 1066 Amsterdam, The Netherlands; 52 Department of Surgery, University of California, San Francisco, San Francisco, California 94143, USA; 53 Cancer Research Laboratory, Faculty of Medicine, University of Iceland, 101 Reykjavik, Iceland; 54 Department of Pathology, University Hospital, 101 Reykjavik, Iceland; 55 Icelandic Cancer Registry, Icelandic Cancer Society, Skogarhlid 8, P.O. Box 5420, 125 Reykjavik, Iceland; 56 Institute for Clinical Medicine, Faculty of Medicine, University of Oslo, 0450 Oslo, Norway; 57 National Genotyping Center, Institute of Biomedical Sciences, Academia Sinica, 128 Academia Road, Sec 2, Nankang, Taipei 115, Taiwan, China; 58 NCCS-VARI Translational Research Laboratory, National Cancer Centre Singapore, 11 Hospital Drive, 169610 Singapore, Singapore; 59 Department of General Surgery, Singapore General Hospital, 169608 Singapore, Singapore

## Abstract

Cancer evolves by mutation, with somatic reactivation of retrotransposons being one such mutational process. Germline retrotransposition can cause processed pseudogenes, but whether this occurs somatically has not been evaluated. Here we screen sequencing data from 660 cancer samples for somatically acquired pseudogenes. We find 42 events in 17 samples, especially non-small cell lung cancer (5/27) and colorectal cancer (2/11). Genomic features mirror those of germline LINE element retrotranspositions, with frequent target-site duplications (67%), consensus TTTTAA sites at insertion points, inverted rearrangements (21%), 5′ truncation (74%) and polyA tails (88%). Transcriptional consequences include expression of pseudogenes from UTRs or introns of target genes. In addition, a somatic pseudogene that integrated into the promoter and first exon of the tumour suppressor gene, *MGA*, abrogated expression from that allele. Thus, formation of processed pseudogenes represents a new class of mutation occurring during cancer development, with potentially diverse functional consequences depending on genomic context.

## Abstract (web summary)

Germline pseudogenes have an important role in human evolution. Here, the authors analyse sequencing data from 660 cancer samples and find evidence for the formation of somatically acquired pseudogenes, a new class of mutation, which may contribute to cancer development.

## Graphical abstract

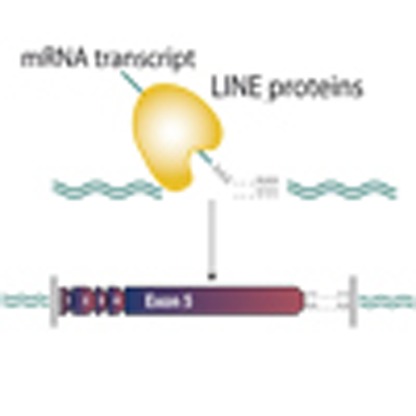

Mutation underpins both the evolution of species and the development of cancer^1^. In the germline, repetitive DNA, such as long interspersed elements (LINE) and Alu repeats, form a sizable proportion of the human genome. LINE elements continue to propagate in the genome through their retrotransposition machinery. Essentially, a functional LINE element, when transcribed and translated by the host machinery, encodes two proteins that co-ordinate reverse transcription of the mRNA template and its integration back into the genome at a distant site from the original element. Insertion of transposable elements has considerably reshaped the human genome over evolutionary time. A role for retrotransposition as a mutational force in somatic cells has been increasingly recognized in the last few years, documented to occur during both normal neurogenesis^2^,^3^,^4^,^5^ and cancer development^6^,^7^,^8^,^9^,^10^.

Germline pseudogenes, representing cDNA copies of mRNA transcripts, are a by-product of LINE-mediated retrotransposition^11^. This copying of DNA sequence through an mRNA intermediate creates several distinctive genomic features of pseudogenes, including the presence of polyA tails, absence of intronic sequence and target-site duplications. In the germline, processed pseudogenes influence evolution through gene duplication, novel exons, gene fusions^12^, sequestration of miRNAs^13^ and antisense transcript production^14^.

Formation of processed pseudogenes has not been systematically studied in cancer. From a screen of 660 cancer samples, we find that somatically acquired pseudogenes are present in 2.6% of cancers, especially lung and colorectal cancers. We find a diverse array of transcriptional consequences, including expression from UTR and intronic insertions, as well as inactivation of transcription from exonic insertions.

## Results

We developed bioinformatic methods to detect somatically acquired processed pseudogenes in massively parallel sequencing data from both targeted exome and genome-wide studies in cancer. Paired-end sequencing reads were aligned to the genome and transcriptome with a view to identifying reads that split exactly across canonical splice sites, were mapped to exons separated by more than the library insert size, or mapped between a pseudogene and its insertion site (Supplementary Fig. 1). To define a putative pseudogene, we required that at least three exons from a single gene were represented in the tumour DNA, with at least two canonical splice junctions directly observed from either split reads or confirmatory capillary sequencing. To establish that pseudogenes were not germline, we analysed sequencing data from the matched normal DNA for the given patient and screened for identical events in other patients. We performed PCR on tumour and matched normal DNA for all predicted pseudogenes with mapped insertion sites, and excluded variants with a positive germline PCR band from this analysis (Supplementary Fig. 2).

**Figure 1:**
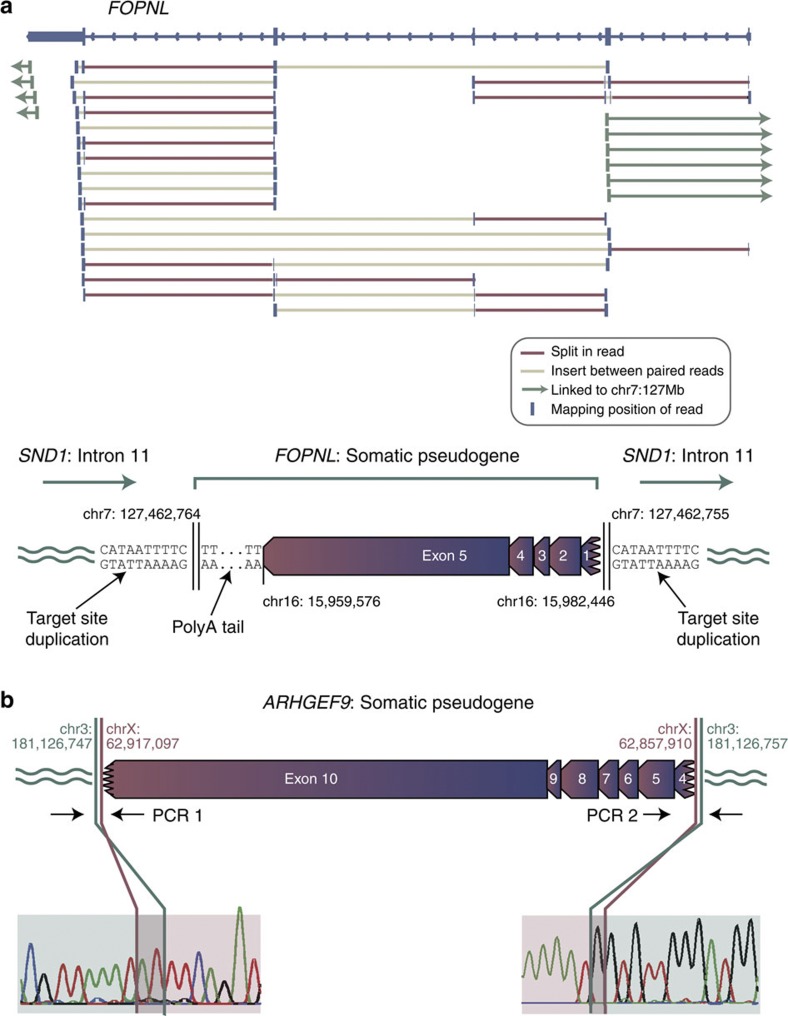
Somatic pseudogenes. (**a**) A somatic *FOPNL* pseudogene in a non-small cell lung cancer. Sequencing reads from high-coverage whole-genome shotgun sequencing of the tumour reveal a series of split reads (red) crossing the four canonical exon–exon splice junctions in the gene. In addition, read pairs map to adjacent exons with an insert size larger than expected (light brown). At either end of the gene, read pairs linking to chr7 could be identified, revealing that the *FOPNL* pseudogene is inserted into intron 11 of the *SND1* gene in the opposite orientation with an intact polyA tail and a target-site duplication of 10 bp. (**b**) A somatic *ARHGEF9* pseudogene in a non-small cell lung cancer. The insertion was confirmed as somatic by PCR (Supplementary Fig. 2) and capillary sequencing across an exon–exon junction and insertion site.

We identified 42 somatically acquired pseudogenes in 14 out of 629 primary cases and 3 out of 31 cell lines sequenced (Table 1, Supplementary Data 1 and Supplementary Table 1). As a typical example, in a lung cancer we identified an insertion of all five exons of the gene *FOPNL,* including a portion of the 5′ UTR, the full coding sequence and the full 3′ UTR, into the eleventh intron of *SND1* in the opposite orientation (Fig. 1a). All four canonical exon–exon junctions of *FOPNL* were crossed by sequencing reads in the tumour DNA, and a polyA tail of at least 50 bp was present at the 3′ end of the pseudogene. No such evidence was seen in the matched germline DNA from this patient or any other, and PCR confirmed the 5′ and 3′ insertion points as somatic. The two insertion points were mapped to base-pair resolution and revealed a target-site duplication of 10 bp that included the motif TTTT at either end of the pseudogene.

**Figure 2:**
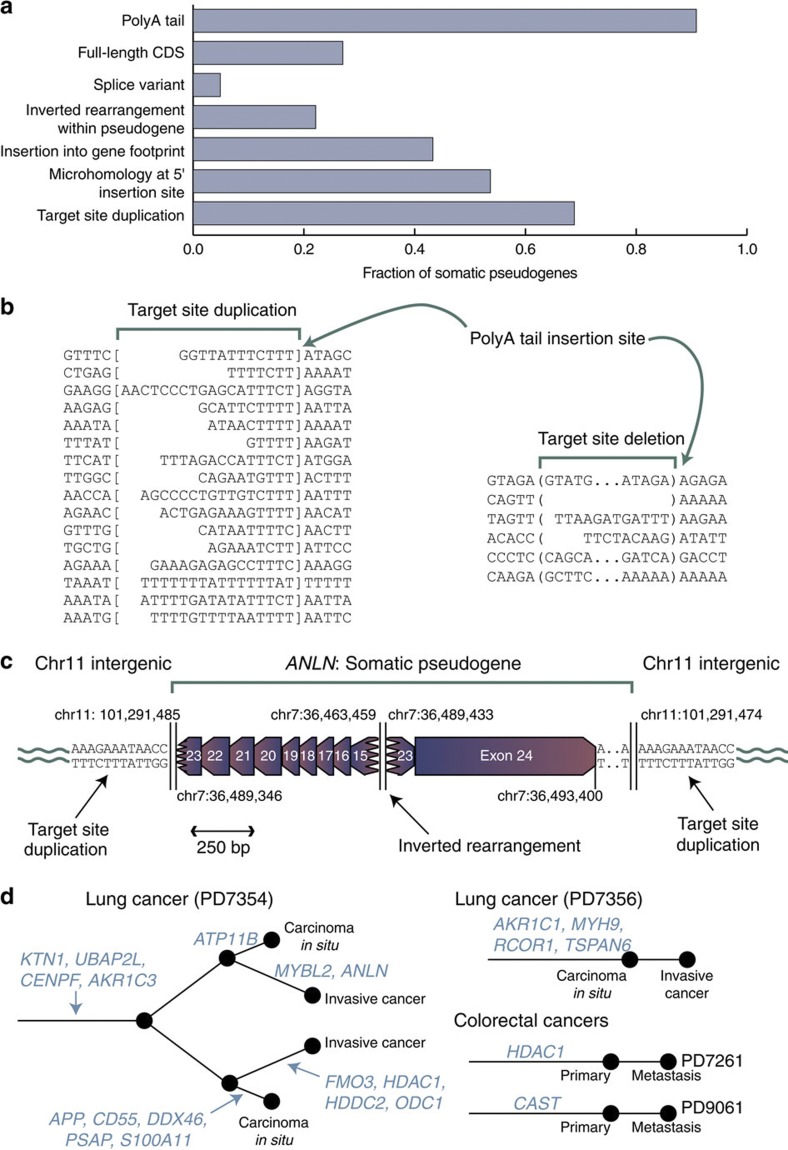
Properties of somatic pseudogenes. (**a**) Histogram showing the fraction of somatic pseudogenes with particular features. (**b**) Sequences of target-site duplications (between square brackets) and adjacent genomic regions, showing that the polyA tail of the somatic pseudogene inserts in the consensus TTTTAA sequence between the TTTT and AA. Target-site deletions were also occasionally seen (deleted sequence between the round brackets). (**c**) An example of an internal inversion in a somatic pseudogene, inserted into intergenic sequence. The insertion was confirmed as somatic by PCR. (**d**) Phylogenetic trees for four patients in whom multiple samples were sequenced, showing at which stage during the evolution of the cancer somatic pseudogenes were acquired.

We observed variations on this theme (Figs 1b and 2a, Supplementary Fig. 3). PolyA tails were usually, but not universally, present, seen in 88% (21/24) pseudogenes with insertion sites fully mapped. Target-site duplications were frequent (67% with insertion sites fully mapped; 16/24), although in five cases target-site deletions occurred. The majority (74%; 31/42) of somatic pseudogenes did not contain the full coding sequence of the gene, as the 5′ end was frequently truncated during reverse transcription and insertion. Two somatic pseudogenes were novel isoforms of their template genes not recorded in the Ensembl database. We did not observe any base substitutions in the somatic pseudogenes, implying that the reverse transcriptase has reasonable fidelity and the template transcripts had not undergone RNA editing.

On the basis of these characteristics, somatic pseudogenes, like their germline counterparts, are probably a product of the reverse transcription and transposition capability of endogenous LINE elements acting on mature mRNA transcripts. Most compelling is the presence of target-site duplications of 8–20 bp, with the point of insertion of the polyA tail almost universally occurring within a TTTTAA or very similar motif (Fig. 2b), which is the classic signature of LINE retrotransposition^15^,^16^. Where we observed target-site deletions at insertion points the sequence showed less consensus, although there was still predilection for AT-rich sequences (Fig. 2b). We also found a somatic pseudogene inserted at the breakpoint of a somatically acquired genomic rearrangement (Supplementary Fig. 4), a process that can occur in the germline^17^.

Interestingly, 21% (9/42) of somatic pseudogenes showed a single inverted rearrangement within the cDNA occurring away from the canonical splice sites of the gene (Fig. 2a,c, Supplementary Fig. 5). Such internal inverted rearrangements are observed in ~8% of L1 LINE elements in the genome^18^ and are thought to occur by a ‘twin priming’ mechanism. Here, not only does the TTTT overhang prime reverse transcription from the polyA tail, but the opposite strand, nicked 10–20 bp downstream, can fold back onto the mRNA transcript internally and prime another cDNA copy. These two partial copies of the mRNA are then resolved by non-homologous end-joining, leading to an inverted rearrangement^19^. Our data are consistent with this mechanism. The internal rearrangements lead to a shuffling of the exon order and direction in the pseudogene, but without duplication of sequence (Fig. 2c). The insertion points of the 5′ end of the inverted pseudogenes showed 1–3 bp of microhomology, consistent with a second priming event. Microhomology of between 1 bp and 4 bp was also usually present at the internal rearrangement breakpoint, indicative of non-homologous end-joining repair.

The 629 primary cases included data from 18 different tumour types (Supplementary Fig. 6, Supplementary Table 2). Somatic pseudogenes were most frequent in non-small cell lung cancer (19%; 5/27 patients) and colorectal cancer (18%; 2/11). The fact that such events occur in colorectal and lung cancers is consistent with the observation of high rates of somatic retrotransposition of LINE elements in these tumour types^7^,^8^,^9^.

For four patients with somatic pseudogenes, we sequenced more than one tumour sample (Fig. 2d). In one patient, we analysed four clonally related samples from two bronchial lesions, both of which evolved from carcinoma *in situ* to invasive cancer. We found somatic pseudogenes common to all four lesions and others unique to a single lesion (Fig. 2d). In another case of lung cancer we found four somatic pseudogenes, all of which were present in both the carcinoma *in situ* and the invasive tumour. Similarly, for two patients with colorectal cancer, we sequenced the primary tumour and a liver metastasis. In both cases, the somatic pseudogene was present in both primary and metastasis. Taken together, these data indicate that somatic pseudogenes can form relatively early in cancer development, before the tumour becomes invasive or metastatic, but can also occur in more advanced stages of disease.

**Figure 3:**
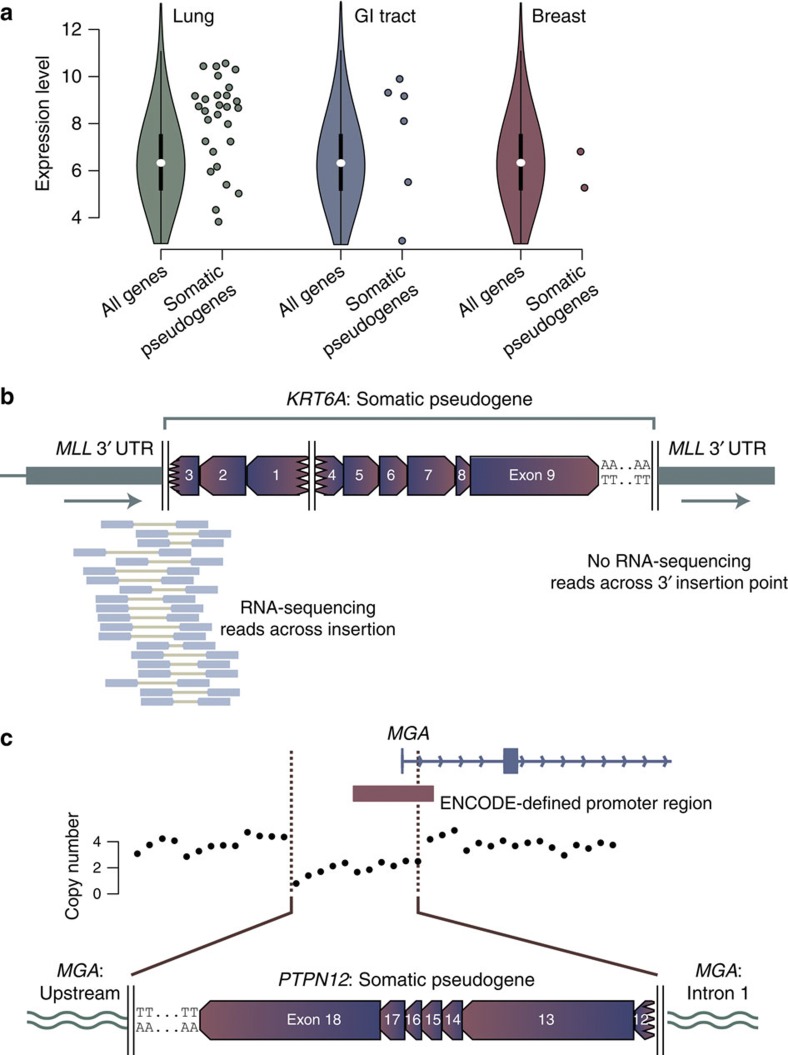
Tissue-specific patterns of somatic pseudogenes. (**a**) Expression of template genes for somatic pseudogenes (individual points) compared with all genes for the most frequently affected organ sites. The violin plot formulation for all genes shows the median (white point), interquartile range (thick black line) and 1.5 × the interquartile range (thin black line). The coloured shapes denote a kernel density plot of the distribution of gene expression levels for all genes. Due to the non-normal distribution, we used Wilcoxon rank-sum tests to test whether expression levels of template genes for somatic pseudogenes were different to that expected. (**b**) RNA-sequencing data showing expression of the *MLL*-*KRT6A* fusion gene. (**c**) Deletion of the promoter and first exon of *MGA* during somatic pseudogene insertion, leading to abrogation of expression from that allele.

Highly expressed transcripts were especially likely to be templates for somatic pseudogenes (Fig. 3a). Overall, 63% of genes acting as the template for somatic pseudogenes were among the top quartile of expressed genes for that tumour type^20^, a statistically significant enrichment (*P*<0.0001, Wilcoxon test). A similar property has been noted for germline pseudogenes^21^. This may explain why we see several examples in which a gene was recurrently retrotransposed as a somatic pseudogene. For example, we found two somatic pseudogene copies of *HDAC1*, one in a colorectal cancer and one in a lung cancer (Supplementary Fig. 7a). Similarly, we see somatic pseudogenes involving two aldo-keto reductase genes, *AKR1C1* and *AKR1C3* (Supplementary Fig. 7b). The aldo-keto reductase proteins activate polycyclic aromatic hydrocarbons in tobacco smoke, a key step in inducing the genotoxicity of these critical carcinogens^22^,^23^. Overexpression of these genes has been documented in lung cancer^24^, which may explain why they recurrently serve as templates for somatic pseudogenes in this disease.

Like any mutational process, the majority of somatic pseudogenes are likely to be passenger mutations, but a few will have functional consequences that may be oncogenic. Somatic pseudogenes could exert functional consequences through many different mechanisms^12^, including fusion gene formation, increased expression of the pseudogene, disruption of a gene at the insertion site, sequestration of miRNAs from the template gene^13^ and production of antisense transcripts^14^. Among the 31 somatic pseudogenes with insertion sites identified, nine were inserted into introns and three into 3′ UTRs. Of these, five were in the same orientation as the host gene, three in the opposite orientation and four had internal inverted rearrangements.

To assess transcriptional consequences of somatic pseudogene insertion, we performed RNA-sequencing on two primary cancers and three cell lines, as well as analysed 20 non-small lung cancers sequenced by TCGA^25^ (Supplementary Table 3). Across these samples, there were 16 somatic pseudogenes, of which 10 were inserted in intergenic regions, three in introns, two in 3′ UTRs and one in the first exon of the *MGA* gene. We found no evidence of expression from somatic pseudogenes inserted in intergenic regions, whereas one of the three intronic insertions was expressed. In this case, a partial *KTN1* pseudogene inserted into the last intron of *PSD3* in a primary squamous cell lung cancer. We saw a clear peak of expression arising from the *PSD3* intron immediately adjacent to the insertion, with aberrantly mapping read pairs aligning to the *KTN1* UTR on one end and the *PSD3* intron on the other end (Supplementary Fig. 8). Of the two insertions into 3′ UTRs, both were expressed. In one, a *KRT6A* pseudogene was inserted into the 3′ UTR of *MLL*, the latter being a well-known fusion gene in leukaemias (Supplementary Fig. 9). The RNA-sequencing data show that the last 1.2 kb of the *MLL* 3′ UTR is lost from the mature transcript and replaced by the somatic pseudogene, since we found paired-end reads spanning the 5′ insertion point but not the 3′ insertion point (Fig. 3b). The 3′ UTR of *MLL* has been shown to regulate transcript levels^26^, a feedback loop that could be disrupted by such a change, although expression of an aberrant transcript does not in itself imply oncogenicity. Similarly, a *KIF18A* pseudogene inserted into the 3′ UTRs of two overlapping genes on opposite strands, *KIAA1967* and *BIN3*. Reads reporting both *KIAA1967-KIF18A* and *BIN3-KIF18A* fusion junctions were found in the RNA-sequencing data (Supplementary Fig. 10).

We also observed that somatic pseudogene insertion could abrogate expression of a target gene at the insertion site. In lung adenocarcinoma cell line NCI-H2009, a *PTPN12* pseudogene caused an 8 kb target-site deletion that removed the promoter and first exon of *MGA* (Fig. 3c). In corresponding RNA-sequencing data, we find only wild-type exon 1 splicing into downstream exons, with no reads linking *PTPN12* to *MGA.* Thus residual expression is derived from the intact *MGA* allele, and loss of the promoter and exon 1 has eliminated expression from the disrupted allele. *MGA* encodes a MAX-interacting protein^27^, with focal deletions and inactivating mutations in lymphoid malignancies^28^,^29^. From a compendium of 7,651 exome sequences^30^, we previously found that *MGA* is a likely tumour suppressor gene on the basis of a statistically significant excess of nonsense mutations (q<10^−6^) especially in lung adenocarcinoma^31^ (Supplementary Fig. 9b).

The diversity, complexity and iniquity of mutational processes operative during the development of cancer have been laid bare by whole-genome sequencing, and here we describe another novel mechanism of somatic mutation. There has been much recent interest in how retrotransposition of repeat elements in somatic cells reshapes the genome during normal brain development and during cancer development^4^,^8^. The formation of pseudogenes in somatic cells represents a companion mutational process, with considerable flexibility in potential mechanisms to alter a cell’s transcriptional activity.

## Methods

### Sequence data

Sequencing data comprised low coverage (2–5 × genome coverage) paired-end sequencing for genomic rearrangements^32^,^33^,^34^, high-coverage (30–40 × ) paired-end shotgun sequencing^35^,^36^ and targeted pull-down and sequencing of the coding exome^37^,^38^ generated at the Wellcome Trust Sanger Institute as described, both published and unpublished, on the Illumina HiSeq platform (Supplementary Table 1). In total, 660 cancer samples (629 primary samples and 31 cell lines) spanning 18 tumour types were analysed (Supplementary Table 2). The identity of cell lines was confirmed by STR testing and were obtained from ATCC. The Cambridgeshire Local Research Ethics Committee approved the studies and all patients gave informed consent.

### Pseudogene detection

Data were aligned to both the reference genome (GRCh37) and the reference Ensembl transcriptome using BWA^39^ and the alignment coordinates from the transcriptome mapping were converted back to genome space. Owing to the different characteristics of exome versus genome data, the analyses of these alignments were optimized to deal with the different data types (Supplementary Fig. 1). It is important to note that, regardless of the analysis method, exome versus genome data are likely to have differential sensitivities for detecting pseudogenes.

Targeted exome data includes less than 2% (50 Mb) of the human genome and provides high depth over exons but does not contain the intronic and intergenic regions where the majority of genomic structural rearrangements lie. Analysis of genomic alignments through our standard structural variant algorithm^35^,^40^ was therefore sufficient for pseudogene detection in these data, as it provided high confidence calls for exon–exon junctions with few calls representing other types of genomic rearrangement. Candidate pseudogenes were required to have at least two apparent deletion events in the same gene, that is, involvement of at least three exons, with breakpoints separated by a distance of at least 500 bp but less than 50 kb, a size range that includes the majority of introns of the human genome^41^. To remove polymorphic germline pseudogenes, groups were excluded if they contained any read pairs from either a matched normal or an unmatched normal panel of nine unrelated individuals. Breakpoints were further excluded if the intervening intron(s) aligned with a deletion annotated in the Database of Genomic Variants, as this indicates a potential germline pseudogene. Transcriptome alignments were used to identify reads that split across exon–exon boundaries by visual inspection in IGV (Integrative Genomics Viewer).

Due to the large size of the genome, the presence of repetitive elements, which tend to be more prevalent in non-coding sequences, and the presence of multiple genomic structural rearrangements in many tumour types, genome data required a custom pipeline to be developed (Perl scripts available on request). After alignment to the transcriptome and conversion of the read mapping coordinates back to genome space, read pairs that are derived from pseudogenes are characterized by a large insert size, due to either reads mapping to adjacent exons, or a split in the CIGAR string where two halves of a read have aligned across a splice junction in the transcriptome. Read pairs were therefore filtered on these characteristics. Retained pairs were required to have an insert size of between 300 bp and 50 kb, two or fewer splits in the CIGAR string with the first and last match being at least 10 bp, and a mapping quality of at least 10. Read pairs passing these criteria were annotated against gene positions at both the 5′ and 3′end of both read 1 and read 2. Read pairs where all four coordinates mapped within the same protein coding gene in Ensembl were retained. The number of supporting read pairs per gene for the tumour, matched normal and an unmatched normal panel of 23 low-depth genomes were calculated and genes with no supporting reads in the normal/normal panel but more than three supporting reads in the tumour were validated by visual inspection in IGV.

### Insertion site mapping

Somatic pseudogenes were best distinguished from contamination of the genomic DNA library by either RNA-sequencing libraries or plasmids containing cDNA expression constructs by mapping the insertion sites of the pseudogene to base-pair resolution. Mappings to the reference genome were used to identify insertion sites using reads mapping between the pseudogene and elsewhere in the genome and/or reads that aligned to one side of the insertion point with soft clipping. Soft-clipped reads were realigned using BLAT, to obtain exact breakpoint coordinates. However, the feasibility of mapping insertion points depended on the data type and the position of the insertion.

In whole-genome shotgun sequencing, the insertion sites were identified for 73% (16/22) of pseudogenes whereas 86% of insertion sites could be mapped in low-depth genomes (Supplementary Data 1). The lower rate of mapping in spite of the higher coverage in whole-genome shotgun sequencing may reflect pseudogene insertions into repetitive sequences in PD7354, which would be unmappable given the short read lengths characteristic of Illumina sequencing.

Alignments to either the genome or transcriptome rarely provided insertion sites for exome data. The majority of somatic pseudogenes consist of the 3′ UTR and varying extents of 5′ truncation. As UTRs are not included in the bait design for exome pull-down, insertion junction sequences are commonly not represented in the bam files of these data. The 5′ end insertion sites were mapped in 29% (2/7) of somatic pseudogenes, with 40% of the remaining pseudogenes being full-length and therefore including a 5′ UTR. The 3′ insertion site could only be mapped in 14% (1/7) of pseudogenes identified in exome data. To assess the relative sensitivity of genome versus exome data for the detection of pseudogenes and capacity to map the insertion site, we sequenced both the exome and whole genome of three cell lines containing somatic pseudogenes to typical depth (Supplementary Table 4). The number of read pairs supporting the presence of a pseudogene through exon-to-exon mappings and the number of discordant read pairs reporting the insertion site for matched exome and genome data were compared. Overall, exome data shows greater sensitivity for splice junctions, whereas genome data are more likely to include the insertion junctions.

### Statistical analysis of recurrent point mutations

To assess whether any of the insertions disrupted tumour suppressor genes at the insertion site, we analysed point mutation calls from 7,651 exomes available from previous publications, TCGA, ICGC and in-house for evidence of a higher than expected rate of inactivating mutation, using an adaptation of the method described in Greenman *et al.*,^42^. This analysis has been described in detail elsewhere^31^.

### PCR validation and capillary sequencing

The somatic status of pseudogenes was confirmed by designing primer pairs between the pseudogene and insertion site (where known) and performing PCR on both tumour and matched normal genomic DNA. DNA for both tumour and matched normal samples was available for 12/16 somatic pseudogene insertion sites predicted from whole-genome (>30 × ) data, all 10 insertion site predictions from low-depth data, and all 3 mapped exome insertions. A total of 92% (11/12) whole-genome predictions validated, with the remaining 8% (1/12) not producing product in either tumour or normal, consistent with PCR/primer failure. All (3/3) exome predictions had confirmed somatic insertion points. Unsurprisingly, low-depth genomes, with no or low coverage sequencing of matching normals, gave a high rate of germline pseudogenes. Overall, 40% (4/10) PCRs showed product in both tumour and normal; 50% (5/10) were somatic and the remaining 10% (1/10) failed to produce product in either tumour or normal. The germline pseudogenes have not been included in this manuscript.

### RNA-sequencing

RNA-sequencing was performed on the three cell lines in which somatically/*in vitro* acquired pseudogenes were identified and two of the primary samples in which somatic pseudogenes were identified.

Total RNA was extracted using Trizol and sequencing libraries prepared by standard Illumina RNA-sequencing of polyA-selected RNA. A 75-bp paired-end sequencing was used and between 24 and 31 Gbp of data were generated per sample. TopHat (v 1.3.3) was used to map reads to the genome. The quality of the RNA-Seq data was assessed using a number of metrics, including absence of 3′ bias and low amounts of ribosomal RNA.

We confirmed the positions, insertion sites and expression of somatic pseudogenes in RNA-Seq using two algorithms (Shlien A., unpublished). First, we looked for discordantly mapped read pairs where one end mapped to the pseudogene and the other near the integration site. We then evaluated grouping of pairs by the consistency of the orientation in which the reads mapped, their position and their overlap with regions of high homology (multi-mapping). Second, we looked for reads that could be partially mapped to the pseudogene and partially mapped to the insertion site (split reads). To do so, we mapped all the RNA-seq data to a transcriptome database containing all known exon–exon junctions. We then shattered and remapped all of the remaining reads into k-mers (13 bp) to an indexed version of the human genome. Mapped fragments were extended one base at a time so long as they maintained a single mapping position. In this way we resolved the breakpoints of the expressed somatic pseudogenes. Note that in the absence of fusion transcript formation, re-expression of the pseudogene from its insertion site was indistinguishable from expression from the template copy.

## Author contributions

S.L.C., A.S., J.M. and A.M. developed algorithms and performed data analysis aided by C.P.P., M.R., L.Y., Y.L., J.M.C.T., H.D., N.B., G.R.B., P.S.T., S.B., S.N.Z., E.P., and V.H.T. Informatics support was provided by K.R., M.S.D., J.W.T. and A.P.B. L.M. and S.O.M. carried out experimental work. I.M. and P.J.C. carried out statistical investigations. C.I.D., T.S., R.G.G., D.M., M.G., N.M., A.M.F., D.B., A.B., J.A.T., D.L., J.S.R.F., D.N.H., S.M.J., P.A.F., M.R.S. and U.M. contributed and verified samples. P.J.C. and S.L.C. wrote the manuscript, with assistance from M.R.S. and U.A.M.

## Additional information

**Accession codes:** Whole-exome sequencing data, whole-genome sequencing data and RNA-sequencing data have been deposited in the European Genome-phenome Archive ( https://www.ebi.ac.uk/ega/) under accession codes EGAD00001000637, EGAD00001000638 and EGAD00001000639, respectively.

**How to cite this article:** Cooke, S. L. *et al.* Processed pseudogenes acquired somatically during cancer development. *Nat. Commun.* 5:3644 doi: 10.1038/ncomms4644 (2014).

## Supplementary Material

Supplementary Figures and Tables Supplementary Figures 1-10 and Supplementary Tables 1-4

Supplementary Data 1 Characteristics of 42 somatic pseudogenes. NTS = non-templated sequence.

## Figures and Tables

**Table 1:** Somatic pseudogenes identified across 660 cancer samples

**Sample**	**Cancer type**	**Pseudogene**	**Exons**	**Full-length**	**Insertion site**	**Target-site dup.**	**Internal inversion**
PD7354c	Lung	*ATP11B*	19–30	No	Unmapped	NA	No
PD7354h	Lung	*ANLN*	15–24	No	Intergenic	12 bp	Yes
PD7354h	Lung	*KTN1*	11–14; 44	No	Intron 15 *PSD3*	7 bp	Yes
PD7354h	Lung	*UBAP2L*	21–26	No	Intergenic	18 bp	No
PD7354h	Lung	*MYBL2*	11–14	No	Intergenic	NA	No
PD7354h	Lung	*AKR1C3*	1–9	No	Unmapped	NA	No
PD7354k	Lung	*APP*	14–16	No	Unmapped	NA	No
PD7354k	Lung	*CD55*	1–11	Yes	Unmapped	NA	No
PD7354k	Lung	*CENPF*	13–20	No	Intergenic	None	No
PD7354k	Lung	*DDX46*	6–12	No	Unmapped	NA	No
PD7354k	Lung	*PSAP*	5–14	No	Unmapped	NA	No
PD7354k	Lung	*S100A11*	1–3	Yes	Upstream *CLMP*	None	No
PD7354r	Lung	*FMO3*	5; 8–9	No	Upstream *TIPIN*	10 bp	No
PD7354r	Lung	*HDAC1*	6–14	No	Intergenic	9 bp	No
PD7354r	Lung	*HDDC2*	1–6	Yes	Rearrangement	None	No
PD7354r	Lung	*ODC1*	8–12	No	Intergenic	5 bp	No
PD7355a	Lung	*GOT1*	5–9	No	Intron 1 *CDH12*	None	No
PD7356c	Lung	*AKR1C1*	6–9	No	Intergenic	14 bp	No
PD7356c	Lung	*MYH9*	28–41	No	Intergenic	10 bp	Yes
PD7356c	Lung	*RCOR1*	5–8; 12	No	Intron 3 *ESR1*	16 bp	Yes
PD7356c	Lung	*TSPAN6*	1–4; 6–8	Yes	Intron 3 *RIT2*	14 bp	No
PD7356i	Lung	*FOPNL*	1–5	Yes	Intron 11 *SND1*	10 bp	No
PD4864b	Lung	*FNTA*	1–9	No	Intergenic	NA	No
PD4864b	Lung	*KRT14*	1–8	No	Unmapped	NA	No
PD4861b	Lung	*ARHGEF9*	4–10	No	Intergenic	None	No
PD4861b	Lung	*HNRNPD*	4–9	No	Intron 1 *RAB8B*	9 bp	No
PD6377a	Gastric	*SLC12A1*	25–27	No	Intron 15 *ADAMTS3*	NA	Yes
PD6384a	Gastric	*POF1B*	1–11; 17	No	Intergenic	NA	Yes
PD6388a	Gastric	*MRPL11*	1–5	Yes	Intron 1 *ABCA13*	15 bp	No
PD7261a	Colorectal	*HDAC1*	1–14	Yes	Intron 1 *RASA2*	NA	No
PD9061a	Colorectal	*CAST*	18–29	No	Intergenic	NA	No
PD6022a	Gastric	*ARF4*	1–6	Yes	Unmapped	NA	No
PD6037a	Cholangiocarcinoma	*CSDE1*	7–16	No	Unmapped	NA	No
PD4226a	Breast	*THUMPD2*	8–10	No	Unmapped	NA	Yes
PD4226a	Breast	*COBL*	10–13	No	Unmapped	NA	No
PD6368a	Chondrosarcoma	*SEP15*	1–5	Yes	3' UTR *WARS2*	NA	No
LB771-HNC	Cell line (H&N)	*KRT6A*	1–3; 4–9	No	3′ UTR *MLL*	17 bp	Yes
LB771-HNC	Cell line (H&N)	*KIF18A*	7–14	No	3′ UTR *BIN3*, *KIAA1967*	None	No
NCI-H2009	Cell line (lung)	*C9orf41*	3–8; 8	No	Intergenic	17 bp	Yes
NCI-H2009	Cell line (lung)	*PTPN12*	12–17	No	Exon 1 *MGA*	None	No
NCI-H2009	Cell line (lung)	*IBTK*	1–29	Yes	Intergenic	None	No
NCI-H2087	Cell line (lung)	*ARPC5*	1–4	Yes	Intergenic	16 bp	No

Insertion sites that could not be mapped may be due to insertion into repetitive sequences or failure of exon capture to include UTRs. H&N, head and neck carcinoma; NA, not available.
