# Supplementary material for: Processed pseudogenes acquired somatically during cancer development
Source: Nat Commun. 2014 Apr 9;5:3644. doi: 10.1038/ncomms4644 (PMC3996531; doi:10.1038/ncomms4644)
Supplement: Supplementary Figures and Tables — Supplementary Figures 1-10 and Supplementary Tables 1-4 [file ncomms4644-s1.pdf]

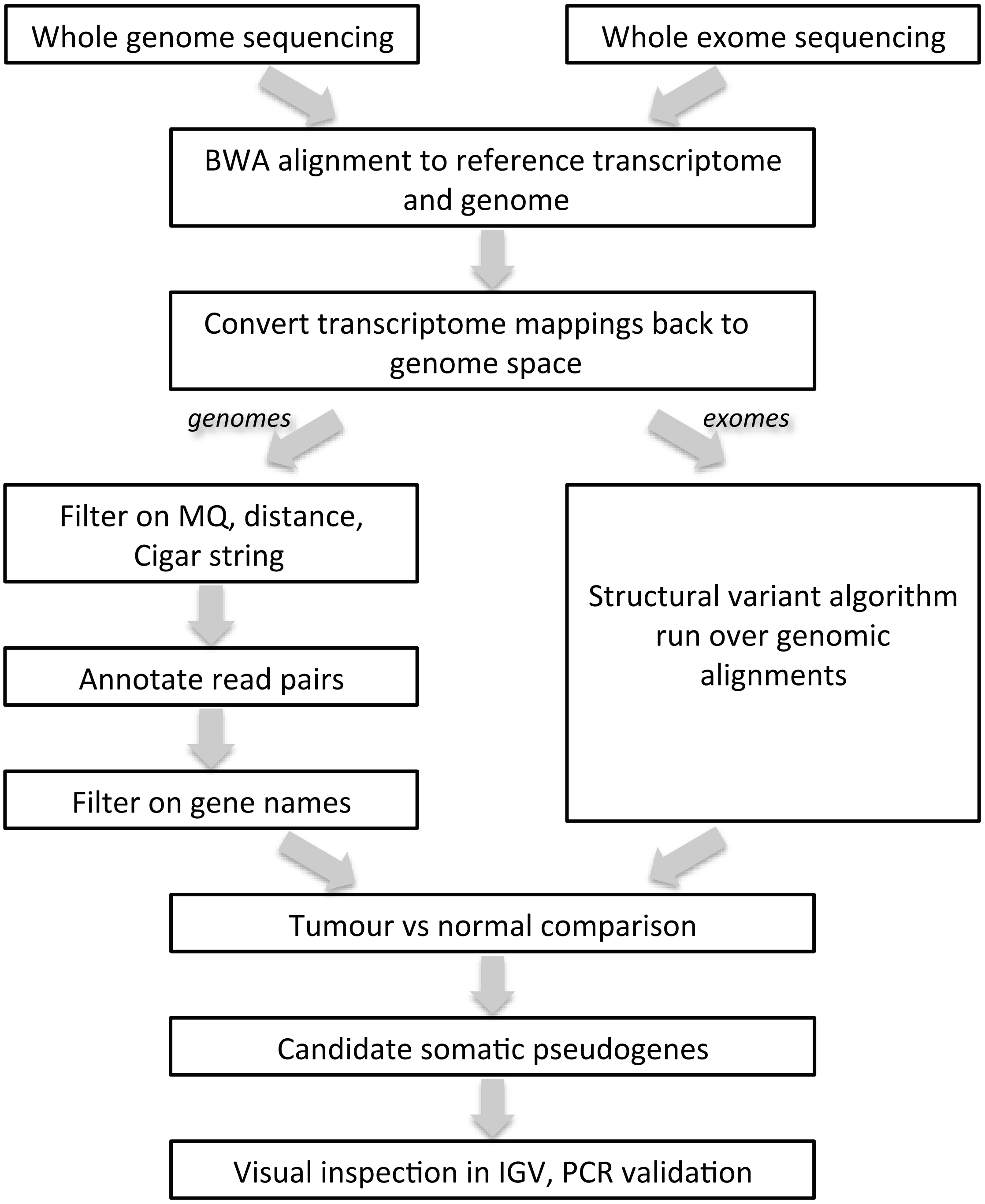

Supplementary Figure 1. Bioinformatic analysis of whole-genome shotgun sequencing and exome pull-down sequencing data.

PCR validations of pseudogenes identified by bioinformatics algorithm

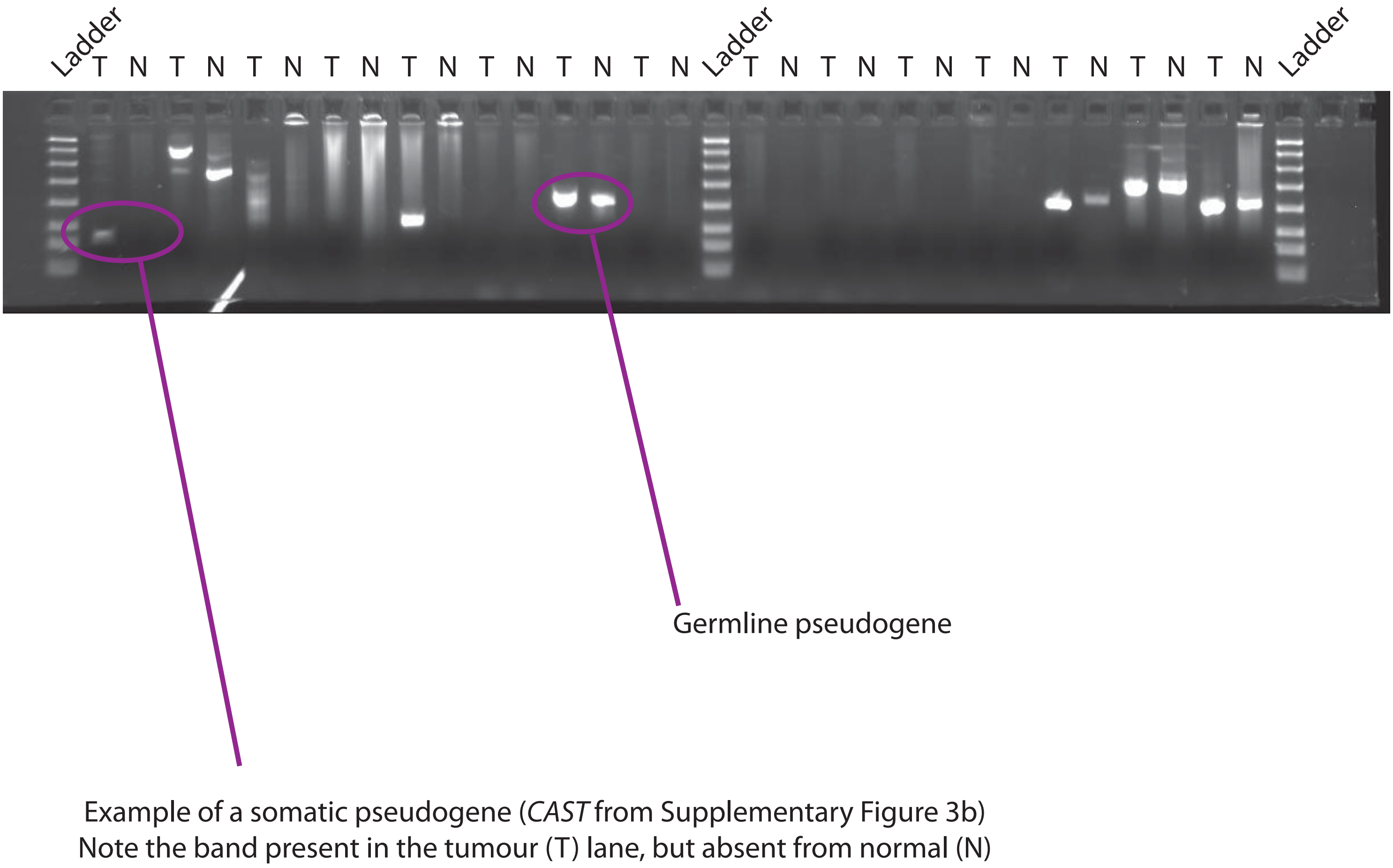

Supplementary Figure 2. PCR validations of somatic pseudogenes. An example gel is shown of PCR validations of somatic pseudogenes. Each PCR was performed on tumour (T) and matched normal (N) DNA, and run on an agarose gel in adjacent lanes. Somatic pseudogenes are recognized by the presence of a band in the tumour lane that is absent from the matched normal DNA, whereas germline pseudogenes have bands present in PCRs from tumour and normal DNA.

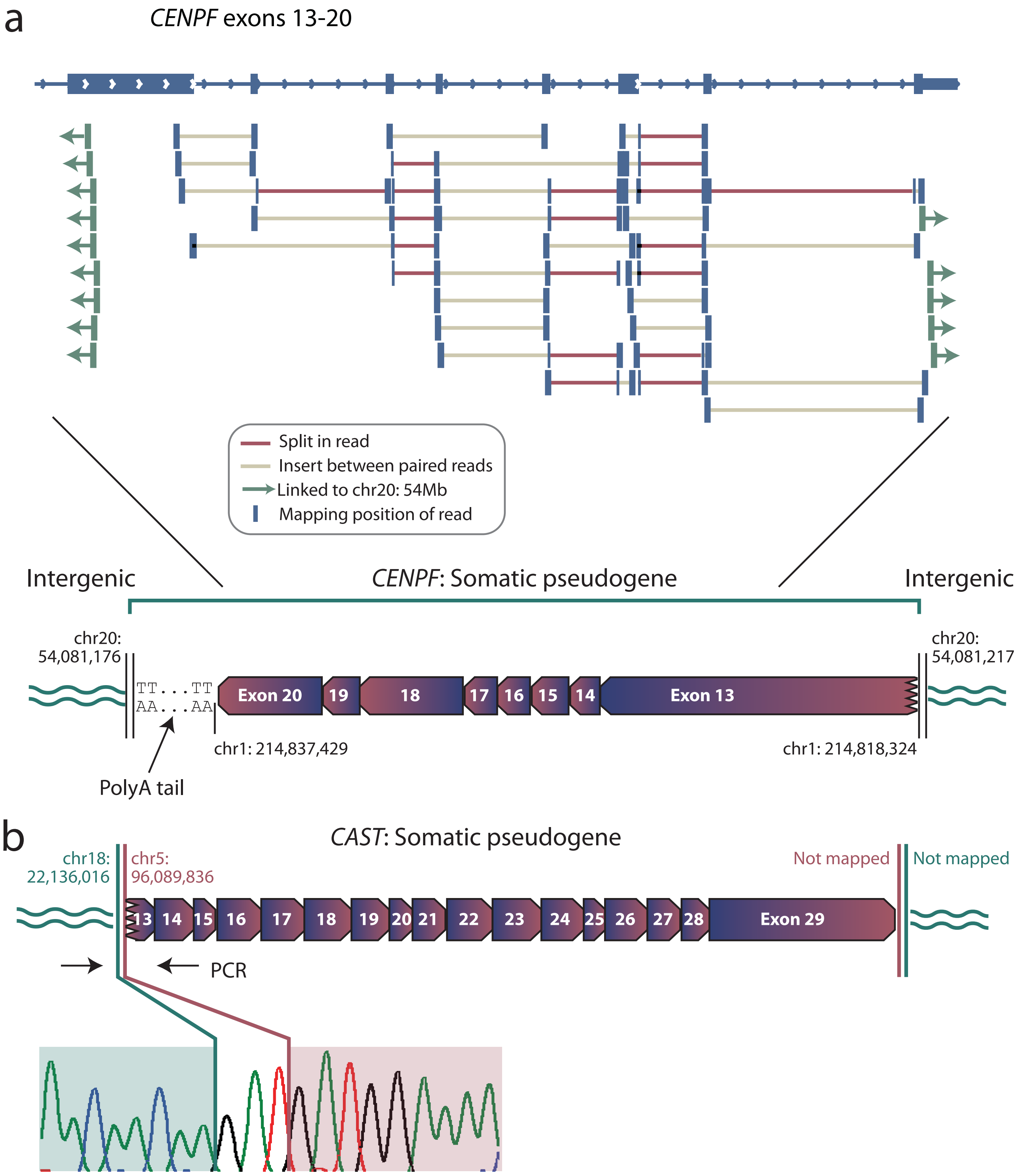

Supplementary Figure 3. Somatic pseudogenes. (a) A somatic *CENPF* pseudogene in a non-small cell lung cancer. Sequencing reads from high-coverage whole genome shotgun sequencing of the tumour reveal a series of split reads (red) crossing the four canonical exon-exon splice junctions in the gene. In addition, read pairs map to adjacent exons with an insert size larger than expected (light brown). At either end of the gene, read-pairs linking to chr20 could be identified, revealing that the *CENPF* pseudogene is inserted into an intergenic region with an intact polyA tail and a target-site deletion of 40bp. (b) A somatic *CAST* pseudogene in a colorectal cancer. The 5′ insertion point was confirmed as somatic by PCR and capillary sequencing across an exon-exon junction and insertion site.

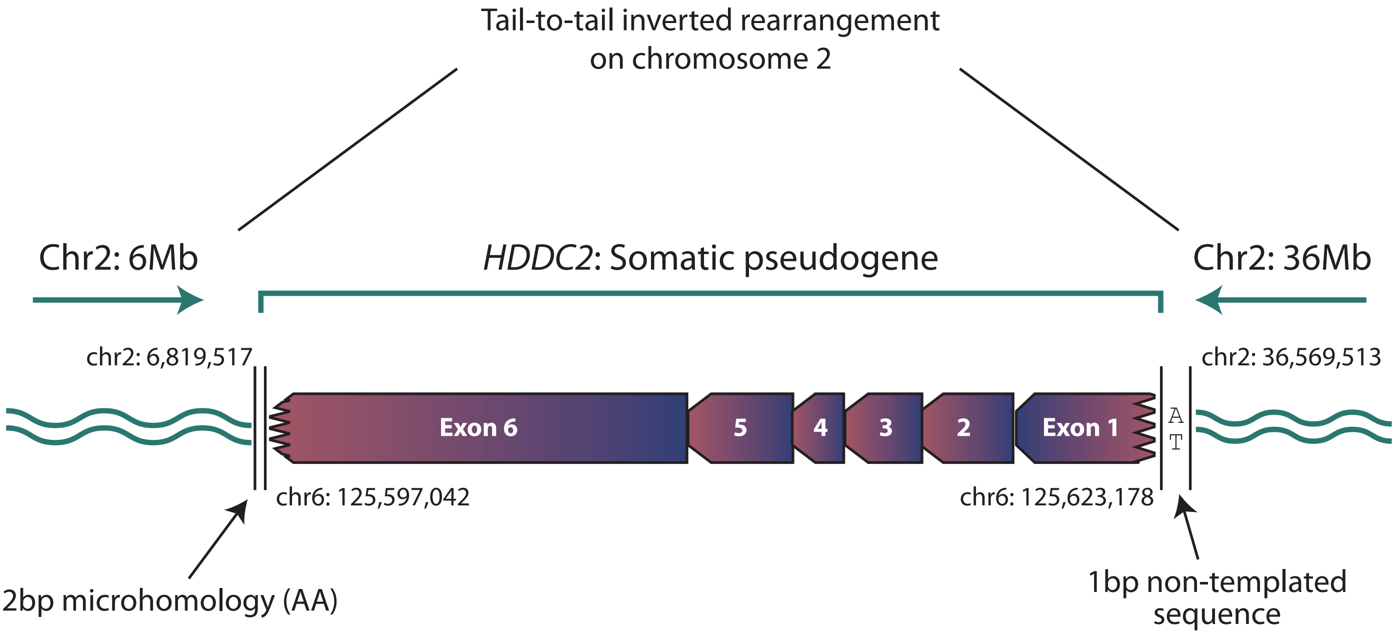

Supplementary Figure 4. A somatic pseudogene was identified in which the 5′ and 3′ insertion points were ~30Mb apart on chromosome 2 and in an inverted orientation. This suggests that the pseudogene was inserted in the breakpoint of a genomic rearrangement during DNA repair.

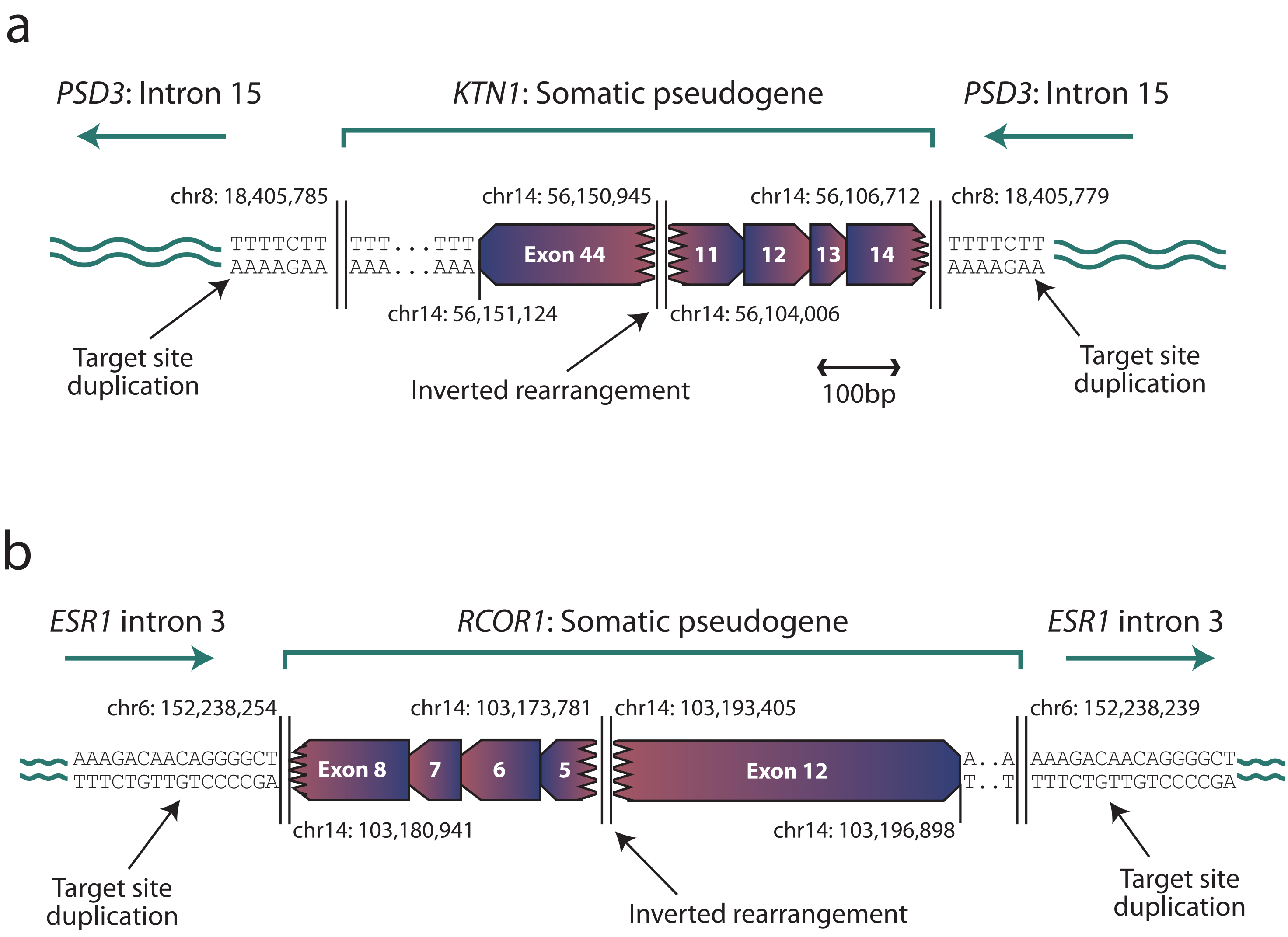

Supplementary Figure 5. Examples of somatic pseudogenes with internal inverted rearrangements.

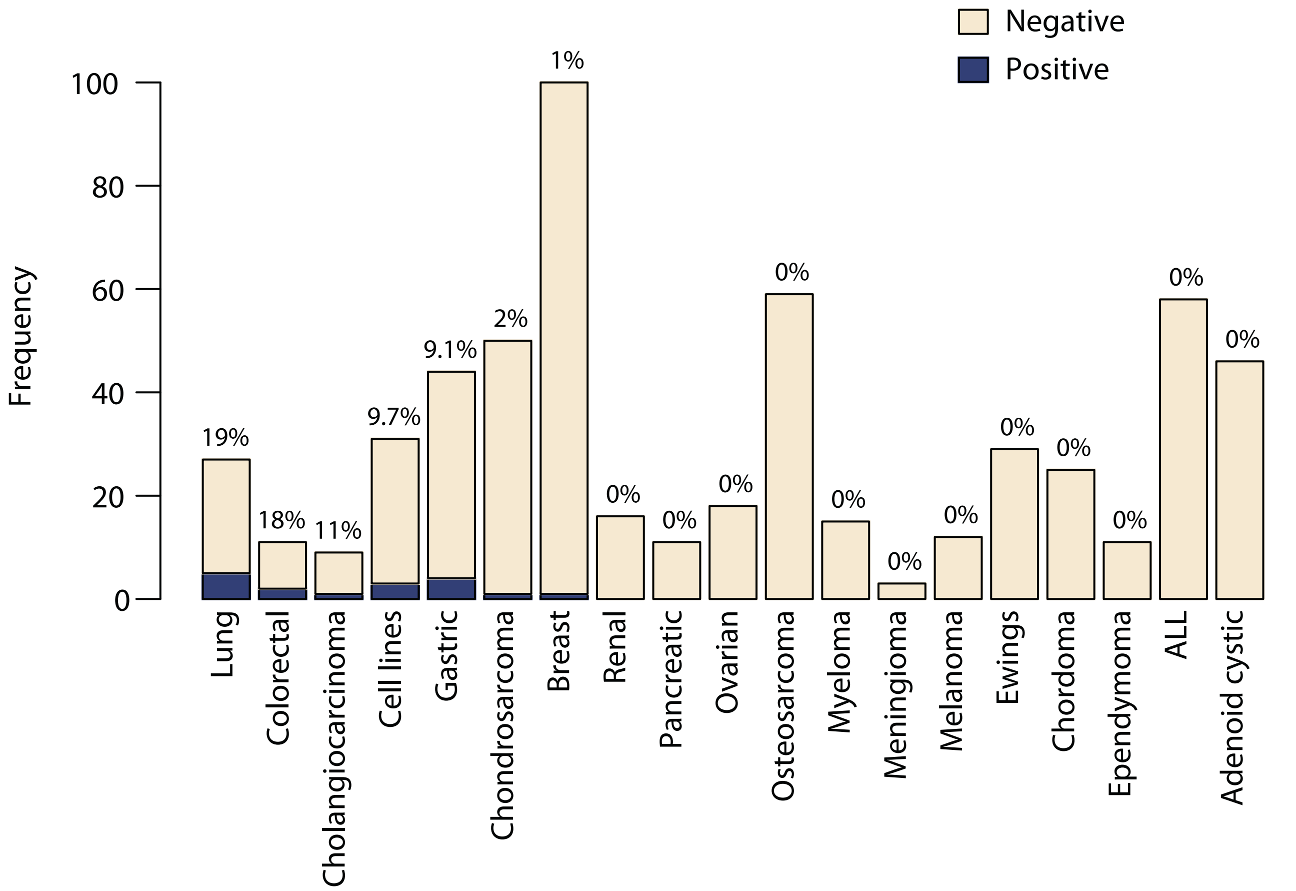

Supplementary Figure 6. Distribution of samples positive (blue) or negative (light brown) for somatic pseudogenes across different tissue types.

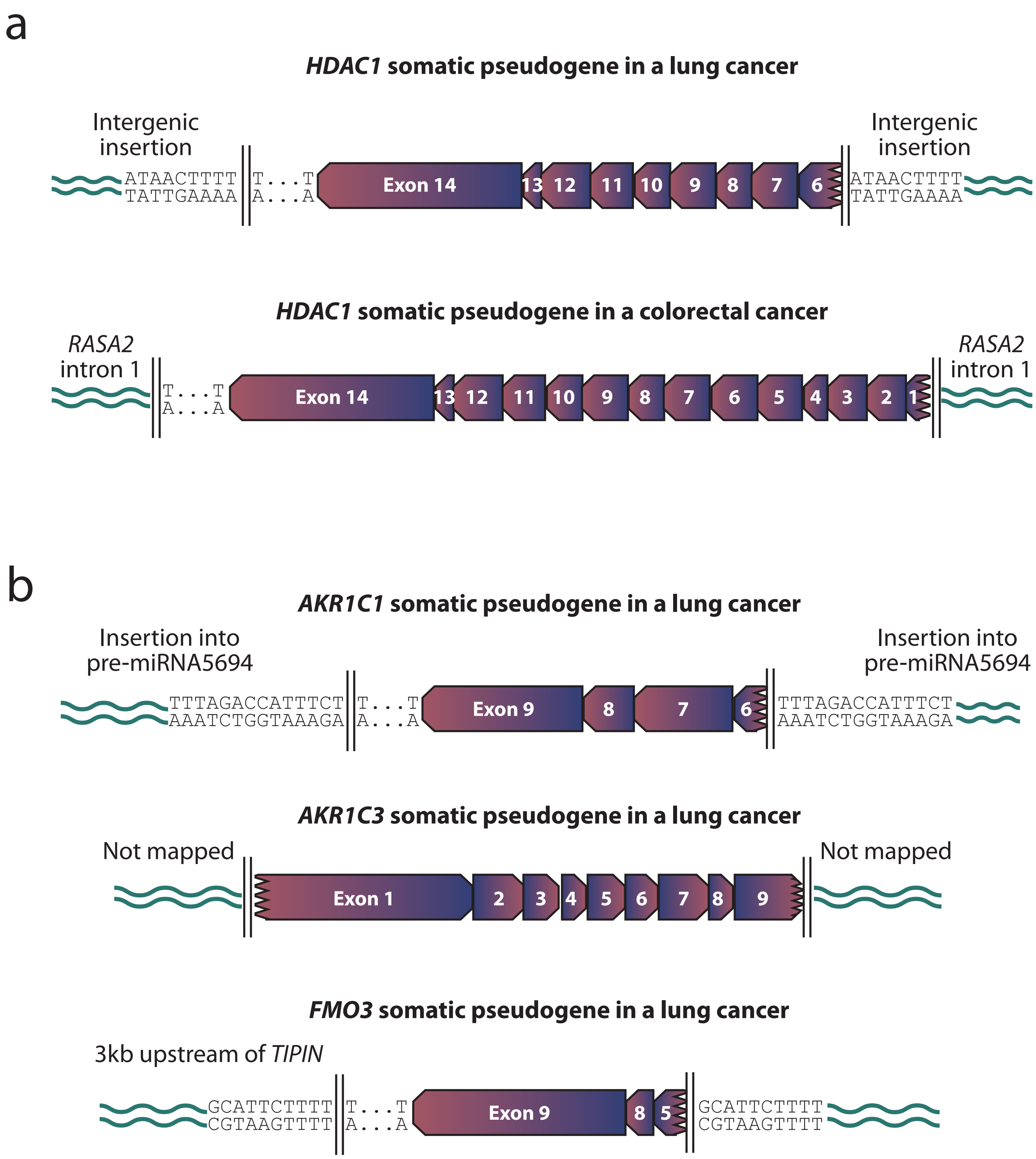

Supplementary Figure 7. Recurrence of somatic pseudogenes. (a) Three somatic pseudogenes involving genes encoding enzymes that metabolise chemicals in cigarette smoke (*AKR1C1*, *AKR1C3* and *FMO3*) observed in lung cancers from smokers. (b) Two somatic *HDAC1* pseudogenes, one full-length in a colorectal cancer and one 5′ truncated in a lung cancer. The different insertion points were confirmed by PCR.

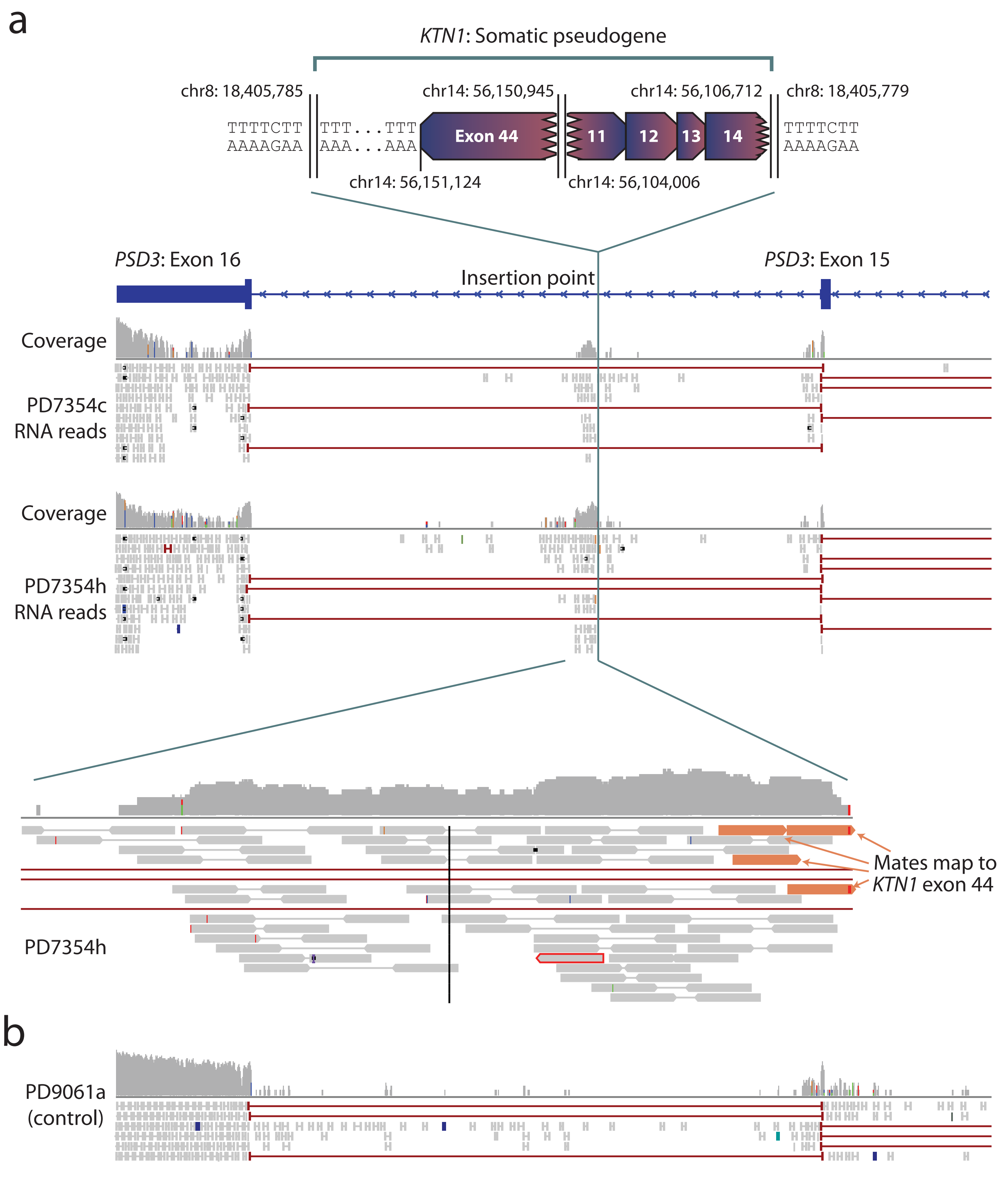

Supplementary Figure 8. Expression of a *KTN1* pseudogene inserted into the last intron of *PSD3* in a primary squamous cell lung cancer. (A) Two RNA samples were available from different time-points during the evolution of this cancer –the somatic pseudogene was present in genomic DNA at both time-points. In the RNA-sequencing data at both time-points (PD7354c and PD7354h), a cluster of read-pairs maps to the *PSD3* intron immediately adjacent to the insertion point (vertical green line). We find four read-pairs (in orange) which align with one end in the *PSD3* intron and the other end in the *KTN1* 3′ UTR, shown in the zoomed-in image. (B) No such expression is seen in another cancer (PD9061a).

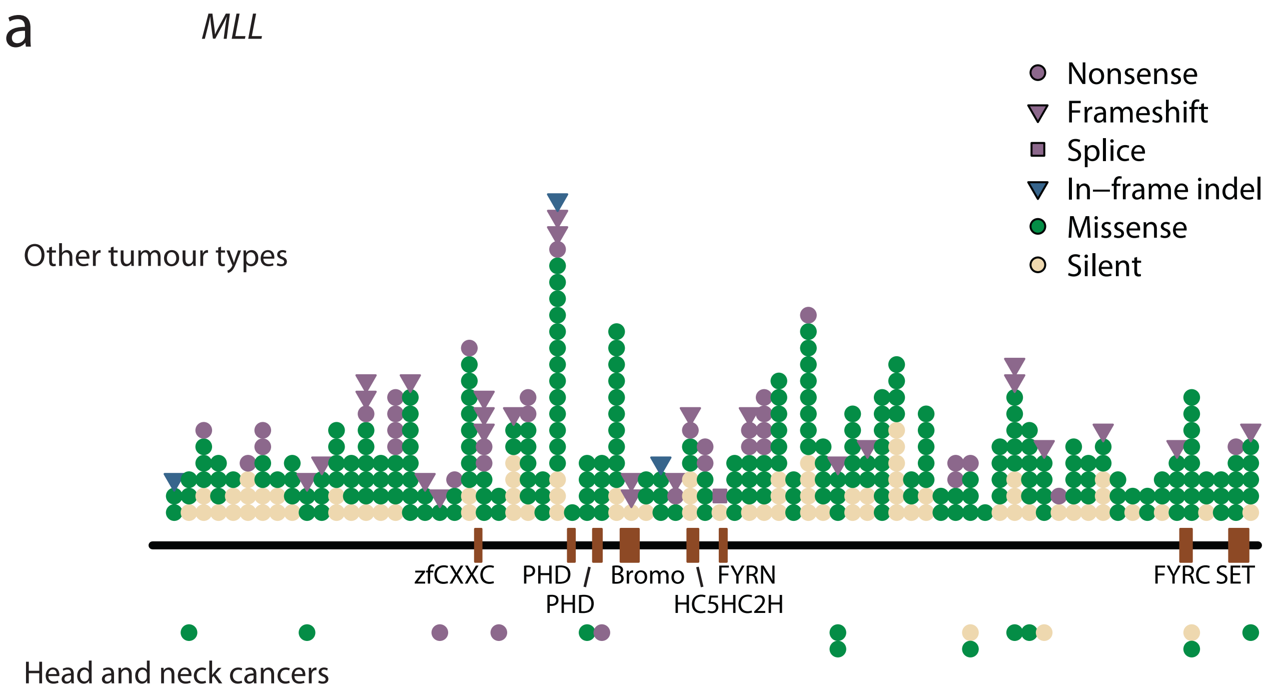

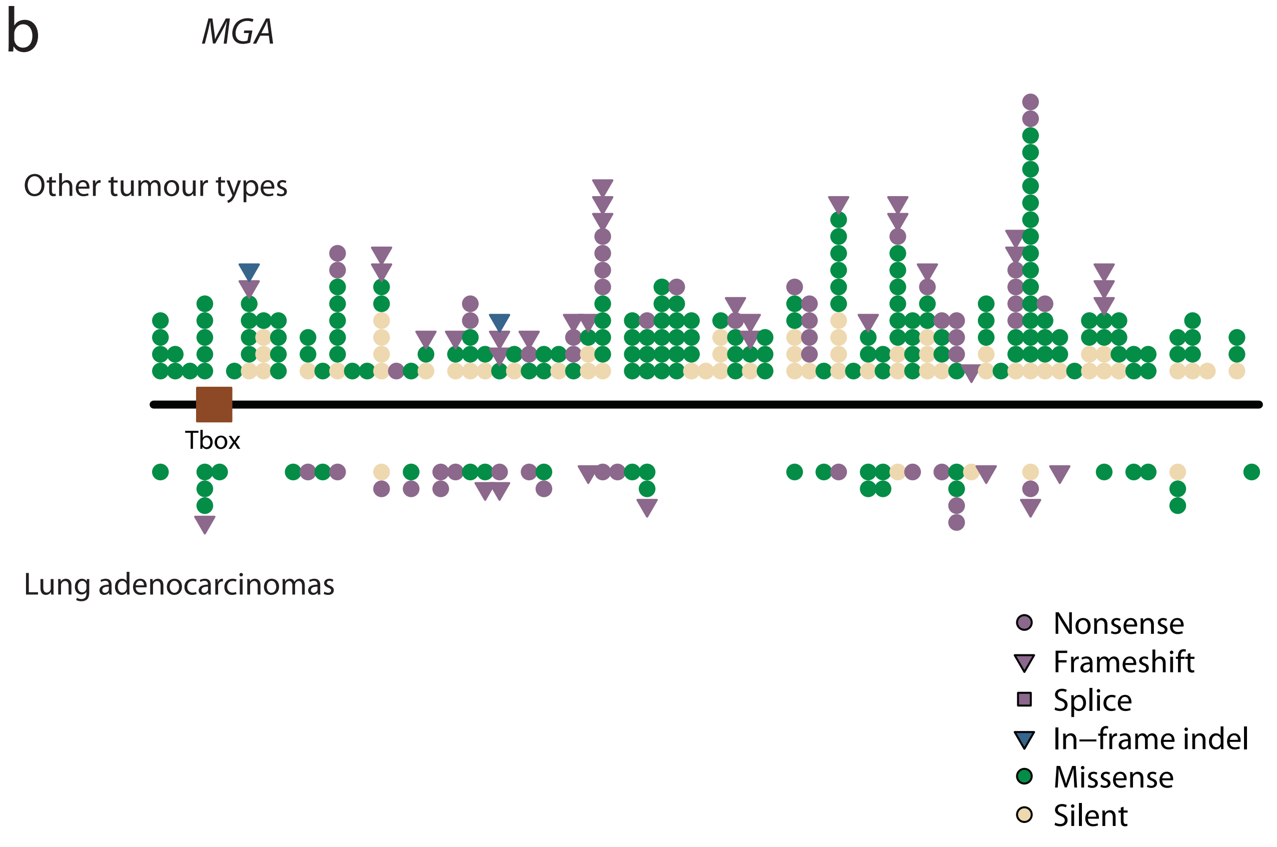

Supplementary Figure 9. Patterns of mutations observed in (a) *MLL* and (b) *MGA* from a compendium of 7,651 publicly available exomes from cancers and matched normal samples. *MGA* shows a statistically significant excess of nonsense mutations (q=4x10-8) by Poisson regression (reference 31, main text) based on 58 nonsense mutations compared to 58 synonymous mutations. This indicates it is a likely tumour suppressor gene.

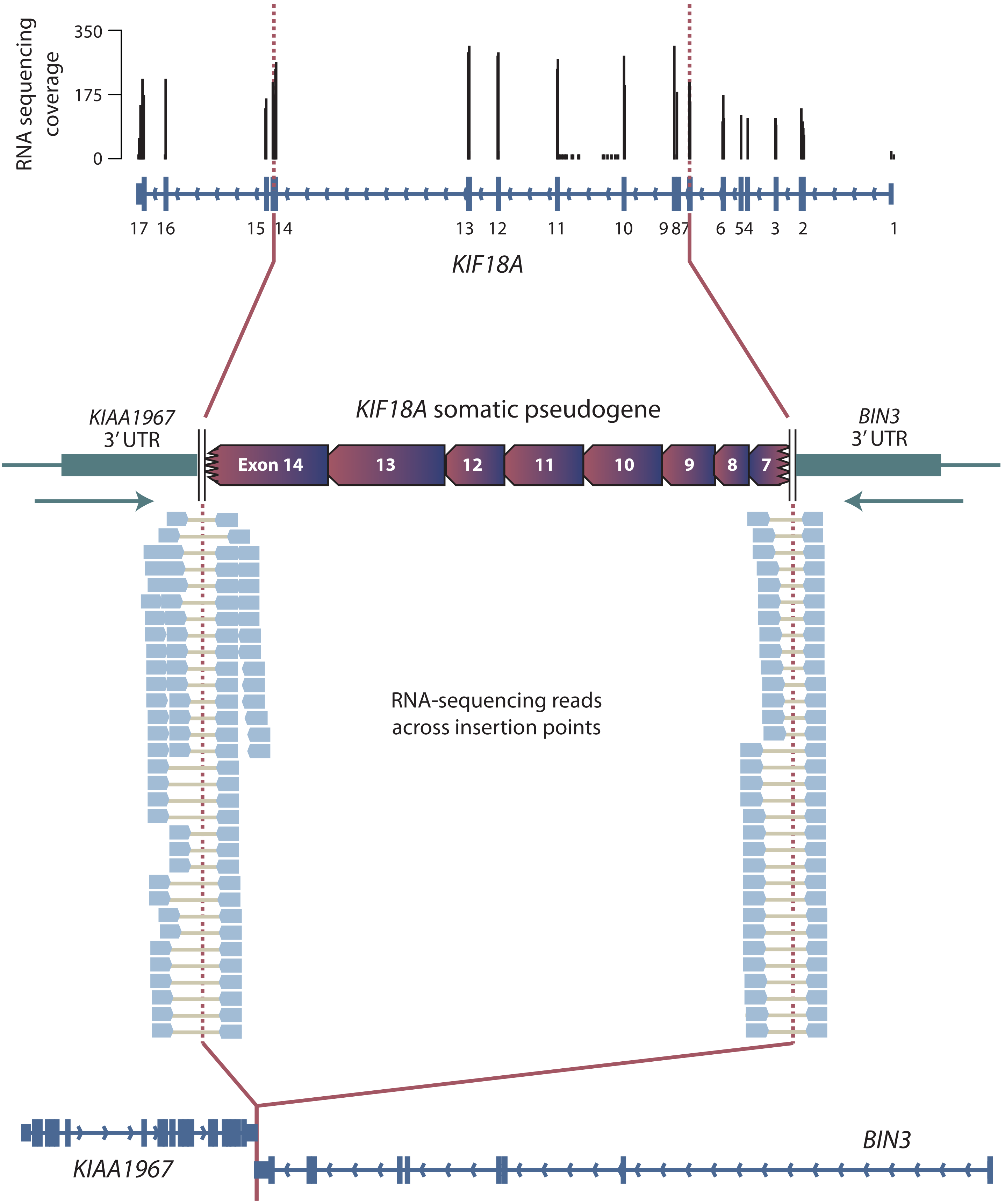

Supplementary Figure 10. RNA-seq of the *KIF18A* somatic pseudogene. Multiple read pairs support expression of the junctions between *KIAA1967-KIF18A* and *BIN3-KIF18A*.

Supplementary Table 1

Sample	Type	Cancer type	Data type	Read length (bp)	Insert size (bp)	Depth	Matched normal depth
PD4226a	Primary	Breast	Exome	76	165	78% at >30x	78% at >30x
PD6037a	Primary	Cholangiocarcinoma	Exome	74	110	52% at >30x	54% at > 30x
PD6368a	Primary	Chondrosarcoma	Exome	75	160	82% at >30x	83% at >30x
PD7261a	Primary	Colorectal	Exome	75	165	79% at >30x	70% at > 30x
PD9061a	Primary	Colorectal	Exome	75	148	76% at >30x	26% at > 30x
PD6022a	Primary	Gastric	Exome	74	120	60% at >30x	60% at >30x
PD6377a	Primary	Gastric	Low depth genome	50	322	4.5x	PCR only
PD6384a	Primary	Gastric	Low depth genome	50	315	5.5x	PCR only
PD6388a	Primary	Gastric	Low depth genome	50	377	5.3x	PCR only
PD7354c	Primary	Lung	Genome	100	313	30.5x	31.9x
PD7354h	Primary	Lung	Genome	100	417	30.4x	31.9x
PD7354k	Primary	Lung	Genome	100	322	30.1x	31.9x
PD7354r	Primary	Lung	Genome	100	304	43.6x	31.9x
PD7355a	Primary	Lung	Genome	100	316	39.0x	31.4x
PD7356c	Primary	Lung	Genome	100	316	31.3x	33.3x
PD7356i	Primary	Lung	Genome	100	298	31.3x	33.3x
PD4861b	Primary	Lung	Low depth genome	50	293	5.7x	2.0x
PD4864b	Primary	Lung	Low depth genome	50	324	6.3x	2.0x
LB771-HNC	Cell line	Head & neck	Exome	75	165	81% at >30x	84% at >30x
NCI-H2009	Cell line	Lung	Exome	75	165	73% at >30x	81% at > 30x
NCI-H2087	Cell line	Lung	Exome	75	165	69% at >30x	80% at > 30x

Supplementary Table 2

Tumour type	Exome (positive)	Genome (positive)	Total (positive)	Prevalence
Adenoid cystic	46 (0)	0	46 (0)	0 %
Acute lymphoblastic leukaemia	58 (0)	0	58 (0)	0 %
Ependymoma	11(0)	0	11 (0)	0 %
Breast	101 (1)	69 (0)	170 (1)	1 %
Cholangiocarcinoma	9 (1)	0	9 (1)	11 %
Chondrosarcoma	50 (1)	0	50 (1)	2 %
Chordoma	25 (0)	0	25 (0)	0 %
Colorectal	11 (2)	0	11 (2)	18 %
Ewing's sarcoma	29 (0)	0	29 (0)	0 %
Gastric	14 (1)	30 (3)	44 (4)	9 %
Lung	0	27 (5)	27 (5)	19 %
Melanoma	12 (0)	0	12 (0)	0 %
Meningioma	3 (0)	0	3 (0)	0 %
Myeloma	15 (0)	0	15 (0)	0 %
Osteosarcoma	59 (0)	15 (0)	59 (0)	0 %
Ovarian	0	18 (0)	18 (0)	0 %
Pancreatic	0	11 (0)	11 (0)	0 %
Renal	16 (0)	0	16 (0)	0 %
Cell lines	31 (3)	0	31 (3)	14%
TOTAL	490 (9)	170 (8)	660 (17)	3 %

Supplementary Table 3

Sample	Gene	Exons involved	Insertion site	5' Insertion Site	Pseudogene into 5' IS	Microhomology / NTS	3' Insertion Site
TCGA-43-3920	SSRP1	3-17	Intergenic	chr17:34,610,045	chr11:57,102,120	4bp microhomology (ATCA)	chr17:34,610,035
TCGA-60-2698	KRT6A	1-4; 4-9	In intron 9 of CTIF	chr18:46,376,578	chr12:52,880,960	polyA tail	chr18:46,376,566
TCGA-60-2713	NOL7	1-8	Intergenic	chr19:21,080,183	chr6:13,615,594	7bp NTS (CAGGCTCT)	chr19:21,079,840
TCGA-60-2722	KRT6A	1-9	Intergenic	chr14:55,576,932	chr12:52,887,038	5bp NTS (GCGAG)	Not mapped
TCGA-38-4630	CNIH4	1-4	In intron 4 of DDO	chr6:110,725,517	chr1:224,544,589	1bp NTS (C)	chr6:110,725,506

Pseudogene into 3' IS	Microhomology / NTS	Target site duplication	Internal rearrangement	Microhomology / NTS	
chr11:57,093,466	polyA tail	ATCAAGCTCTT			
chr12:52,884,672	1 bp microhomology (G)	GGCCTTCCTTTTT	Inverted exons 1;4	1bp microhomolog	52884672 to 52886571
chr6:13,621,127	polyA tail	200bp duplication			
Not mapped	Not mapped	Not mapped			
chr1:224,563,688	polyA tail	TCCTTTTGTCTT			

Supplementary Table 4

Pseudogene	Data type	Depth	Insertion junctions	splice junctions
KRT6A	Exome	81% at >30x	0	121
KRT6A	Genome	41x	13	48
KIF18A	Exome	81% at >30x	9	90
KIF18A	Genome	41x	27	29
C9orf41	Exome	73% at >30x	0	155
C9orf41	Genome	46x	38	54
PTPN12	Exome	73% at >30x	12	171
PTPN12	Genome	46x	13	43
IBTK	Exome	73% at >30x	0	636
IBTK	Genome	46x	24	244
ARPC5	Exome	69% at >30x	4	55
ARPC5	Genome	40x	21	23
